# The telomeric DNA damage response occurs in the absence of chromatin decompaction

**DOI:** 10.1101/gad.294082.116

**Published:** 2017-03-15

**Authors:** Aleksandra Vancevska, Kyle M. Douglass, Verena Pfeiffer, Suliana Manley, Joachim Lingner

**Affiliations:** 1 Swiss Institute for Experimental Cancer Research, School of Life Sciences, Ecole Polytechnique Fédérale de Lausanne (EPFL), 1015 Lausanne, Switzerland;; 2 Institute of Physics, Laboratory of Experimental Biophysics, EPFL, 1015 Lausanne, Switzerland

**Keywords:** telomeres, DNA damage response, chromatin compaction, STORM

## Abstract

Telomeres are specialized nucleoprotein structures that protect chromosome ends from DNA damage response (DDR) and DNA rearrangements. The telomeric shelterin protein TRF2 suppresses the DDR, and this function has been attributed to its abilities to trigger t-loop formation or prevent massive decompaction and loss of density of telomeric chromatin. Here, we applied stochastic optical reconstruction microscopy (STORM) to measure the sizes and shapes of functional human telomeres of different lengths and dysfunctional telomeres that elicit a DDR. Telomeres have an ovoid appearance with considerable plasticity in shape. Examination of many telomeres demonstrated that depletion of TRF2, TRF1, or both affected the sizes of only a small subset of telomeres. Costaining of telomeres with DDR markers further revealed that the majority of DDR signaling telomeres retained a normal size. Thus, DDR signaling at telomeres does not require decompaction. We propose that telomeres are monitored by the DDR machinery in the absence of telomere expansion and that the DDR is triggered by changes at the molecular level in structure and protein composition.

## Abstract (precis)

In this study, Vancevska et al. apply stochastic optical reconstruction microscopy (STORM) to measure the sizes and shapes of functional human telomeres of different lengths and dysfunctional telomeres that elicit a DNA damage response. They show that depletion of TRF2, TRF1, or both triggered a DDR without a concomitant telomere decompaction, demonstrating that the telomeric DDR is not linked to chromatin decompaction.

Telomeres protect chromosome ends from degradation, DNA rearrangements, and DNA damage signaling, which are seen at DNA double-strand breaks (de Lange 2009; Denchi and Sfeir 2016). The repetitive DNA sequences at human telomeres consist of several kilobases of double-stranded TTAGGG repeats ending in a single-stranded 3′ overhang of 100–300 nucleotides. Telomeres are associated with a large number of proteins that mediate their function (Dejardin and Kingston 2009; Grolimund et al. 2013; Bartocci et al. 2014). The shelterin proteins are the main constituents of telomeres, comprising six specialized proteins (de Lange 2005). Among these, both TRF1 and TRF2 bind directly as dimers to the double-stranded portion of telomeric DNA. In contrast, POT1 forms a dimer with TPP1 and binds to the single-stranded G-rich telomeric DNA (Baumann and Cech 2001). TIN2 and Rap1 associate indirectly with telomeres—TIN2 through interactions with TRF1, TRF2, and TPP1, and Rap1 through interactions with TRF2. Shelterin proteins are essential for mediating telomere functions. In particular, TRF1 is required for efficient replication of the TTAGGG repeats by the DNA replication machinery (Sfeir et al. 2009). TRF1 recruits the BLM helicase, which sustains replication, and TPP1/POT1, which represses ATR kinase signaling (Zimmermann et al. 2014). In the absence of TRF1, replication forks stall, and telomeres obtain a fragile phenotype. Stalled replication forks accumulate ssDNA, which, when bound by replication protein A, recruits ATRIP–ATR to initiate a DNA damage response (DDR) (Zou and Elledge 2003). This can explain how TRF1-depleted telomeres activate the ATR checkpoint kinase in S phase.

The shelterin TRF2 protects chromosomes from end-to-end fusions by nonhomologous end-joining (NHEJ) and suppresses activation of the ATM checkpoint kinase (van Steensel et al. 1998; Denchi and de Lange 2007). When telomeres become critically short, they fail to recruit sufficient TRF2, leading to the activation of a DDR and cellular senescence. Thus, the uncapped telomeres, as DNA double-strand breaks, are sensed and bound by the Mre11–Rad50–Nbs1 (MRN) complex, recruiting and activating the ATM kinase (Uziel et al. 2003; Lee and Paull 2005). ATM then phosphorylates various substrates, culminating in the DDR cascade.

Telomere-bound TRF2 simultaneously inhibits ATM kinase (Karlseder et al. 2004) and the propagation of DNA damage signaling downstream from ATM (Okamoto et al. 2013). In parallel, MRN recruitment and ATM activation at telomeres may be prevented through t-loops. In t-loop structures, the telomeric 3′ overhang is tucked into the double-stranded part of the telomere (Griffith et al. 1999) and may therefore hide the ends of chromosomes from the DNA damage machinery. T-loops were first detected when analyzing psolaren cross-linked telomeric DNA that had been purified from human or mouse cells (Griffith et al. 1999). More recently, when analyzing cross-linked chromatin spreads in vitro by stochastic optical reconstruction microscopy (STORM), t loops were found at ∼20% of telomeres with varying strand invasion points (Doksani et al. 2013). Intriguingly, depletion of TRF2 caused loss of t loops. Thus, TRF2-dependent suppression of DDR and t-loop formation are correlated. A very different alternative model was proposed recently in which loss of TRF2 would lead to an up to 10-fold decompaction (decrease in density) of telomeric chromatin, rendering telomeres accessible to DDR factors that would otherwise be excluded (Bandaria et al. 2016). Within this model, activation of ATR signaling upon TRF1 depletion was also explained by chromatin decompaction rather than the accumulation of ssDNA at stalled replication forks in S phase as discussed above. The telomere decompaction model was based on data obtained with superresolution microscopy on human cells in which TRF1, TRF2, or other shelterin components were depleted.

Here, we applied STORM superresolution fluorescence microscopy to study telomere structure (Rust et al. 2006). With STORM, we can determine the positions of individual fluorescent probes on a telomere with precision on the order of 10 nm by stochastically switching the fluorophores between fluorescent and dark states. A STORM measurement on a single telomere yields a cluster of fluorophore position estimates (known as localizations) from which structural properties of the telomere, such as its size and shape, were calculated. We depleted TRF1 and TRF2 to assess their roles in telomere compaction and used a large field of view (FOV) flat illumination microscope setup to capture a large number of telomeres (>900 per condition) with high image quality (Douglass et al. 2016). By costaining telomeres with the DNA damage markers 53BP1 and γH2AX, we were able to unequivocally distinguish telomeres eliciting a DDR from intact telomeres. Our results reveal that the vast majority of DDR-positive telomeres does not differ in size from DDR-negative telomeres, excluding telomere decompaction from being generally associated with the DDR.

## Results

### STORM imaging of human telomeres

**Figure 1.**
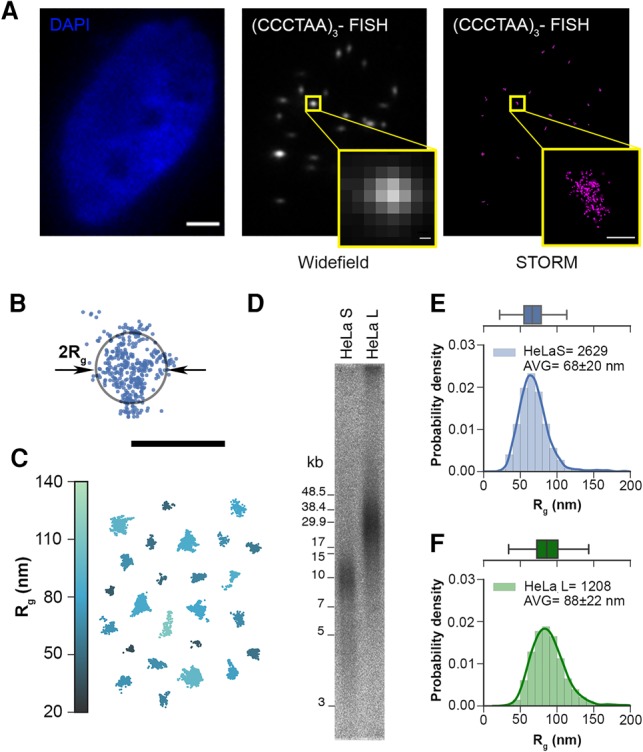
Human telomeres are heterogeneous in length, size, and shape. (*A*) Wide-field fluorescence images of the HeLa cell nucleus stained with DAPI (*left*; bar, 3 µm) and telomeres labeled with telomeric (CCCTAA)_3_-A647 fluorescence in situ hybridization (FISH) probe (*middle*) and STORM image of telomeres (*right*). Enlarged *insets* show that telomeres are smaller than the diffraction limit. Bar, 0.15 µm. (*B*) The signal from a single telomere is a cluster of fluorescent molecule position estimates known as localizations. Its size is determined by the radius of gyration (*R*_g_) of the localizations (bar, 0.2 µm), which is the root-mean-square distance of the localizations from the cluster's center of mass. (*C*) Several clusters of localizations from HeLa L (HeLa cells with long telomeres) telomeres illustrating their heterogeneity in shape and *R*_g_ (color-coded in the vertical bar). (*D*) Telomere restriction fragment analysis of telomere length of isogenic HeLa S (HeLa cells with short telomeres) and HeLa L cells used for STORM imaging displays the length heterogeneity of HeLa telomeres. (*E*) Distribution of *R*_g_ for (CCCTAA)_3_-FISH-labeled samples of HeLa S telomeres. The solid line on the histogram plot is the kernel density estimate of the distribution, and the solid vertical lines in the box mark the quartiles. Whiskers mark the range of the distribution, excluding outliers. (*F*) The same as *E* but for HeLa L telomeres.

To visualize the TTAGGG repeats of human telomeres, we hybridized fixed HeLa cells with a PNA oligonucleotide (5′-CCCTAA-3′)_3_ probe that was labeled at its 5′ end with the fluorescent dye Alexa fluor 647. Imaging was performed with a custom-built microscope capable of performing STORM on 10–30 cells simultaneously, facilitating the acquisition of large data sets and better ensuring sufficient sampling over the sample heterogeneity. The mean localization precision of fluorophores was 10 nm in the *X* and *Y* directions (Supplemental Fig. S1). As expected, wide-field imaging showed telomeres as diffraction-limited spots (Fig. 1A). However, STORM imaging resolved individual fluorophores, forming clusters of localizations corresponding to telomeres. To ensure that every cluster corresponded to a telomere, the localizations were overlaid with wide-field images, and the data were filtered to reject groups of signals that did not correspond to an image of a telomere, had a very low number of localizations (<50), or were not properly clustered. Many telomeres adopted roughly an ovoid structure, but the heterogeneity of shapes suggested a considerable plasticity of telomeres (Fig. 1C; Supplemental Fig. S2).

We assessed telomere sizes by computing each cluster's radius of gyration (*R*_g_), which is the root-mean-square distance between the localizations and the cluster center. Our measured *R*_g_ values correlate well with another measure of size, the convex hull area (Supplemental Fig. S1). Unlike the convex hull, however, which uses only localizations at the extreme edges and assumes that cluster outlines have no concavities, *R*_g_ uses every localization in the cluster to determine telomere size and makes no assumptions on telomere shape. We compared the telomeres of two isogenic HeLa strains, termed HeLa S (HeLa cells with short telomeres) and HeLa L (HeLa cells with long telomeres), in which the average telomere length was 11 kb and 33 kb as determined by telomere restriction fragment length analysis (Fig. 1D). The long telomeres in Hela L were obtained upon overexpression of the catalytic subunit of telomerase hTERT together with RNA moiety hTR (Cristofari and Lingner 2006; Grolimund et al. 2013). The average *R*_g_ of HeLa S was 68 nm, and the average *R*_g_ of HeLa L 88 nm (Fig. 1E,F). Therefore, longer telomeres showed a larger *R*_g_, as expected. The spread in the distributions is consistent with the measured heterogeneity of telomere lengths. Considering that the volume of a sphere increases with the third power of the radius, we estimated that HeLa L telomeres have a slightly higher density (1.4×) than HeLa S (see the Materials and Methods).

### Telomere sizes of shelterin-depleted telomeres

**Figure 2.**
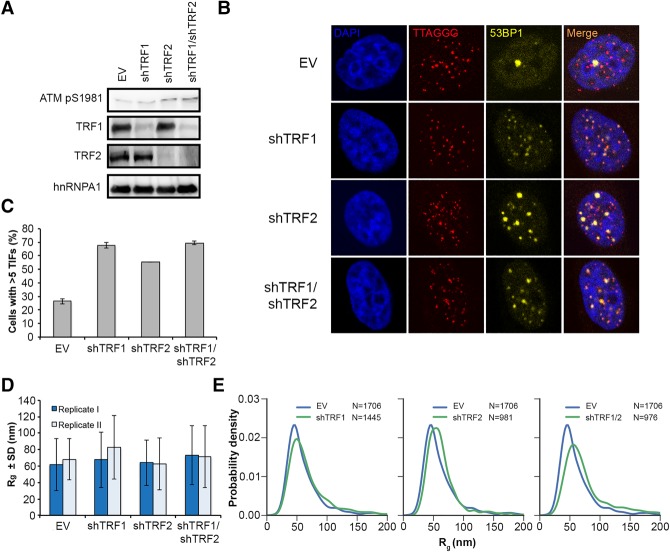
Depletion of shelterin proteins TRF1 and TRF2 does not affect telomere size in HeLa S cells. (*A*) Western blot analysis of TRF1, TRF2, hnRNPA1, and ATM pS1981 in HeLa cells transfected with the indicated shRNAs (shTRF1, shTRF2, and shTRF1/shTRF2) or empty vector (EV). (*B*) Representative images for detection of 53BP1 at telomeres in HeLa cells transfected with the indicated shRNAs or empty vector. Immunofluoresence (IF) for 53BP1 (yellow) was combined with telomeric (CCCTAA)_3_-FISH (red), and the DNA was stained with DAPI. (*C*) Quantification of the number of cells containing more than five telomere dysfunction-induced foci (TIFs), detected as in *B*. Data represent the mean of two independent experiments ± SD (>130 cells per condition per experiment). (*D*) Average *R*_g_ of telomeric (CCCTAA)_3_-FISH-labeled samples obtained by analysis of STORM data. Data represent the mean *R*_g_ (in nanometers) of two independent experiments ± SD (>900 telomeres per condition per experiment). (*E*) Representative distributions of *R*_g_ of telomeric (CCCTAA)_3_-FISH-labeled samples obtained by analysis of STORM data.

To study the roles of the shelterin proteins TRF1 and TRF2 in telomere size maintenance, we depleted TRF1 and TRF2 upon expression of shRNAs in HeLa cells from transiently transfected vectors (Fig. 2). Alternatively, we transiently overexpressed a mutant version of TRF2 (TRF2ΔBΔM) that is dimerization-competent but DNA-binding-deficient and instrumental in titrating off endogenous TRF2 from telomeres (van Steensel et al. 1998) or used siRNAs targeting TRF1 (Supplemental Fig. S3). The depletion of TRF1 and TRF2 was confirmed on Western blots (Fig. 2A; Supplemental Fig. S3A,D). A loss of function was indicated by the accumulation of the activated and phosphorylated form of the checkpoint kinase ATM (Fig. 2A). The accumulation of the DNA damage marker 53BP1 in foci that colocalized with telomeres indicated that the damage occurred at telomeres (Fig. 2B,C). We then determined the *R*_g_ in control cells and TRF1-depleted, TRF2-depleted, and TRF1/2-double-depleted cells. Strikingly, the radii had similar mean values and similar variances (Fig. 2D,E). Therefore, upon strong reduction of shelterin proteins, the telomeric chromatin did not change its compaction in a significant manner. However, it must be noted that, in depletion experiments, only a fraction of telomeres elicit a DDR, as evidenced by the accumulation of the DDR marker 53BP1 at only a subset of telomeres (Fig. 2C; Supplemental Fig. S3B,E). Therefore, these experiments did not rule out specific changes at DDR-active versus DDR-inactive telomeres.

### Telomeric DDR in the absence of decompaction

**Figure 3.**
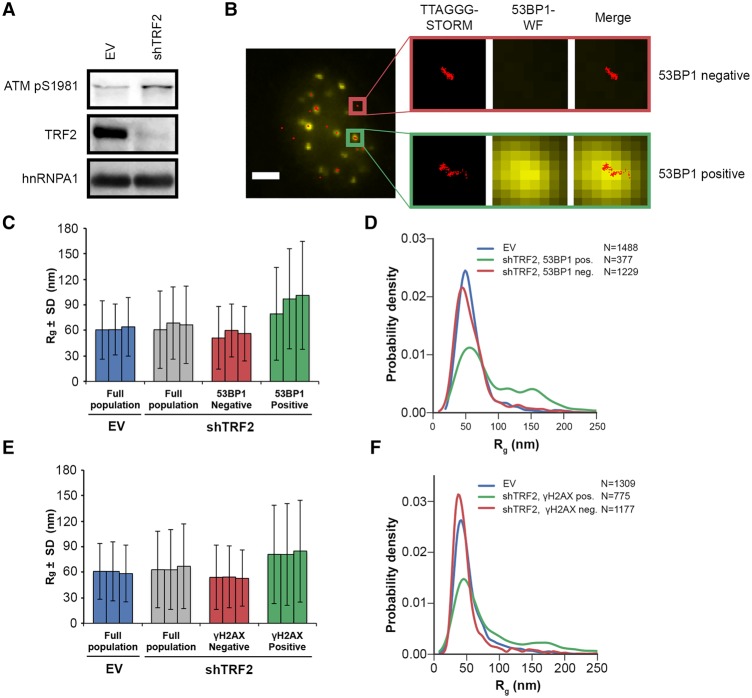
Selection of DDR-positive telomeres of shTRF2-depleted HeLa S cells with two markers (53BP1 and γH2AX) reveals an increase in telomere size in only a small subset of telomeres. (*A*) Western blot analysis of TRF2, hnRNPA1, and ATM pS1981 in HeLa cells transfected shTRF2 plasmids. (*B*) Representative images for detection of 53BP1 at telomeres in HeLa cells transfected with shTRF2 plasmids. IF for 53BP1 (yellow; *middle* panel, wide-field [WF] image) was combined with telomeric (CCCTAA)_3_-FISH (red; *left* panel, STORM image) in order to use the wide-field image of 53BP1 as a selection marker for DDR-positive telomeres. One 53BP1-positive and one 53BP1-negative telomere are enlarged. The same procedure was also performed for selection of γH2AX-positive telomeres. Bar, 4 µm. (*C*) Average *R*_g_ of telomeric (CCCTAA)_3_-FISH-labeled and 53BP1-IF-labeled samples obtained by analysis of STORM data. Data represent the mean *R*_g_ (in nanometers) of three independent experiments ± SD (>900 telomeres per condition per experiment). (*D*) Representative distributions of *R*_g_ of telomeric (CCCTAA)_3_-FISH-labeled and 53BP1-IF-labeled samples obtained by analysis of STORM data. (*E*) Average *R*_g_ of telomeric (CCCTAA)_3_-FISH-labeled and γH2AX-labeled samples obtained by analysis of STORM data. Data represent the mean *R*_g_ (in nanometers) of three independent experiments ± SD (>900 telomeres per condition per experiment). (*F*) Representative *R*_g_ distributions of telomeric (CCCTAA)_3_-FISH-labeled and γH2AX-labeled samples obtained by analysis of STORM data.

To identify and compare the sizes of DDR-positive and DDR-negative telomeres, we costained telomeres with antibodies against either 53BP1 or γH2AX, both of which can serve as DDR markers (Fig. 3; Supplemental Fig. S4). The average *R*_g_ of DDR-positive telomeres was slightly larger than that of DDR-negative telomeres. However, this shift in the mean value was due to a small subset of DDR-positive telomeres that had a considerably larger *R*_g_ (∼10% of control telomeres had an *R*_g_ of >100 nm, whereas 37% of 53BP1-positive telomeres had an *R*_g_ of >100 nm) (Fig. 3D). However, the vast majority of DDR-positive telomeres had an *R*_g_ that was indistinguishable from DDR-negative telomeres. This indicates that chromatin decompaction is not required for the telomeric DDR.

**Figure 4.**
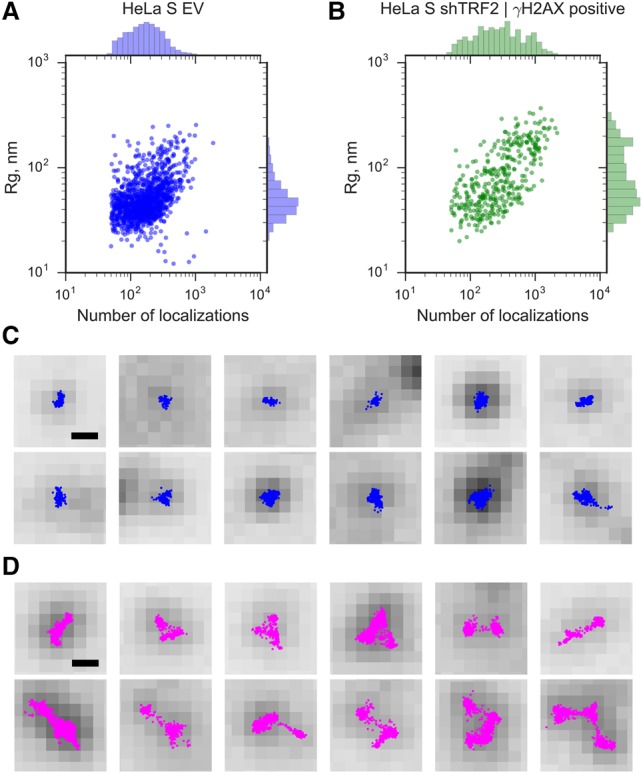
An increase in the size of DDR-positive telomeres of shTRF2-depleted HeLa S cells is accompanied by an increase in the number of localizations. (*A*) *R*_g_ (in nanometers; distribution shown at the *right*; log scale) as a function of the number of localizations (distribution is shown at the *top*; log scale) in HeLa S cells transfected with empty vector controls (EV). Each dot represents a single telomere. (*B*) *R*_g_ as a function of the number of localizations in HeLa S cells transfected with shTRF2 plasmids. The telomeres shown were selected as DNA damage-positive using γH2AX as a DDR marker. Each dot represents a single telomere. (*C*) Randomly selected STORM images overlaid with wide-field images of γH2AX-positive telomeres that have *R*_g_ values <80 nm. Bar, 250 nm. (*D*) Randomly selected STORM images overlaid with wide-field images of γH2AX-positive telomeres that have *R*_g_ values >80 nm. Bar, 250 nm.

Efficient depletion of TRF2 is known to lead to telomere associations and chromosome end-to-end fusions in addition to eliciting a telomeric DDR. We therefore suspected that the small subset of DDR-positive telomeres with larger *R*_g_s contained more telomeric DNA and possibly corresponded to telomere associations. Consistent with this, we observed a positive correlation between the number of localizations and *R*_g_ (Fig. 4A), including a population of DDR-positive telomeres with a higher number of localizations and larger *R*_g_s compared with the control (Fig. 4B). For the longer telomeres in HeLa L, we also observed a larger number of localizations (mean *n* = 412) than in HeLa S (mean *n* = 299), although their ratio was not in proportion with their average lengths, which differed by a factor of three. Finally, fluorescence intensity in fluorescence in situ hybridization (FISH) experiments has been correlated with telomere length in numerous studies (Poon and Lansdorp 2001). Altogether, these analyses suggest that the small subset of DDR-positive telomeres in TRF2-depleted cells that had a larger *R*_g_ contained more telomeric DNA. Since depletion of TRF2 during a short time period does not induce telomere length changes (Supplemental Fig. S5), this suggests that the larger telomeres were due to telomere–telomere associations. This was supported by inspection of their shapes, which further indicated larger deviations from the ovoid shapes that were seen in DDR-positive telomeres with a near-average *R*_g_ as well as control telomeres (cf. Fig. 4C,D and Supplemental Fig. S2). However, these experiments could not fully exclude that a higher number of localizations could be due to increased probe accessibility.

### Telomere size measurements by FISH and anti-TRF1 immunofluorescence (IF) are consistent

**Figure 5.**
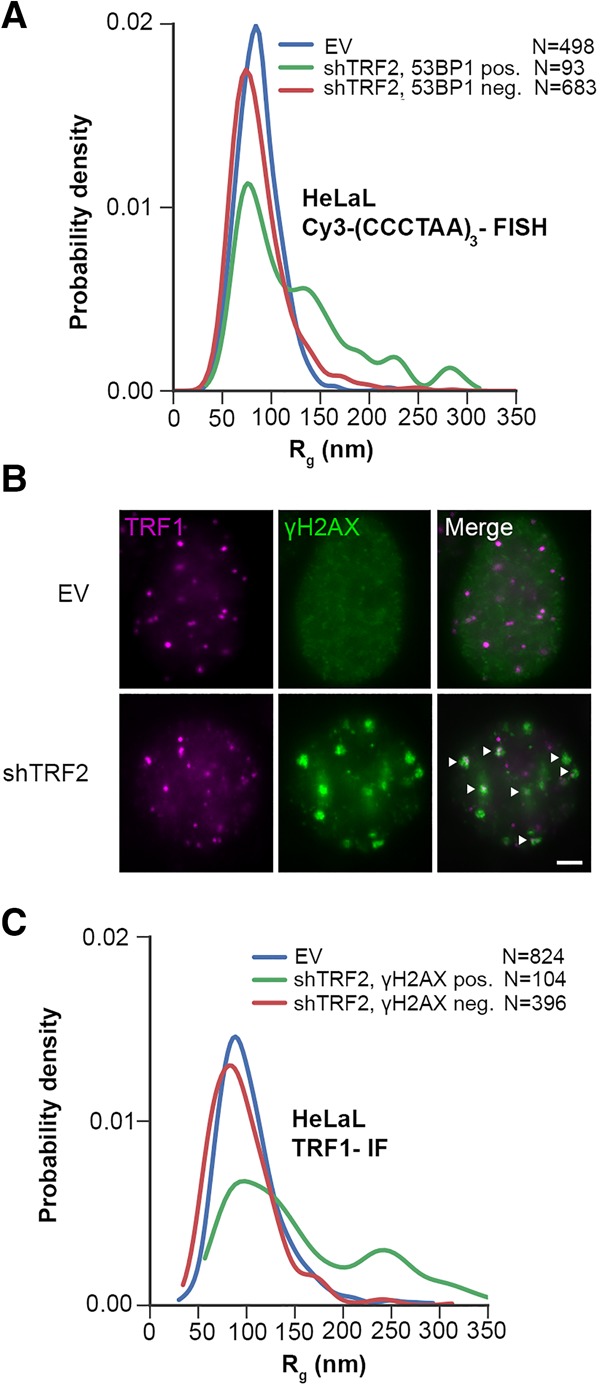
Measurements of HeLa L telomere sizes, labeled by FISH or anti-TRF1 IF, are consistent. (*A*) Representative distribution of *R*_g_ of telomeric (CCCTAA)_3_-FISH-labeled and 53BP1-IF-labeled HeLa L samples obtained by analysis of STORM data. (*B*) Representative images for detection of γH2AX and TRF1 at telomeres in HeLa L cells transfected with shTRF2 or empty vector (EV) plasmids. Bar, 3 µm. Both TRF1 (purple) and γH2AX (green) were detected by IF. Arrows indicate colocalization of telomeres, with γH2AX used for discrimination of DDR-positive and DDR-negative telomeres. (*C*) Representative distribution of *R*_g_ of telomeric TRF1-IF-labeled and γH2AX-IF-labeled HeLa L samples obtained by analysis of STORM data.

To further corroborate our analysis, we compared telomeres that were stained with FISH probes with telomeres that were stained by indirect IF with affinity-purified polyclonal antibodies raised against TRF1 (Fig. 5). The experiments were carried out with HeLa L cells with telomeres of an average length of 33 kb, as they gave a good signal over noise (Fig. 5B). The average *R*_g_ of HeLa L was 88 nm when telomeres were labeled by FISH (Figs. 1F, 5A) and 103 nm when labeled by IF against TRF1 (Fig. 5C). The slightly larger *R*_g_ obtained with IF can be explained by the sizes of primary and secondary antibodies that will place the fluorescent label at an offset distance from TRF1-bound telomeres and therefore lead to an apparent size increase (Lambert and Waters 2017).

We also compared the *R*_g_ distributions of telomeres in HeLa L that had been depleted for TRF2. For FISH and IF, the DDR-negative telomeres had a distribution similar to that of empty vector cells. For DDR-positive HeLa L telomeres, we observed with both methods a major peak in the size distribution that was indistinguishable from that of nondepleted cells and a smaller subset of DDR-positive telomeres with larger *R*_g_s as compared with the control. Therefore, the analyses by FISH and IF are consistent. Furthermore, the telomeres of HeLa L and HeLa S responded similarly to the depletion of TRF2. In both cases, the great majority of telomeres eliciting a DDR did not increase in size.

## Discussion

In this study, we applied STORM to study the shape and size of human telomeres. Using a large FOV illumination system known as FIFI (Douglass et al. 2016), we were able to simultaneously sample multiple cells that differed in their telomeric states. The determined telomere sizes demonstrate that telomeric DNA is compacted when assembled as chromatin in cells. A B-DNA double helix of 11,000 base pairs (bp) has a calculated length of 3650 nm. The measured *R*_g_ of 68 nm for 11,000 bp of telomeric chromatin suggests a compaction in length of <27-fold. Our measurements are in agreement with previous studies on mouse and human telomeres (Doksani et al. 2013; Bandaria et al. 2016). Our analysis reveals that the volume elements occupied by human telomeres can be approximated by ovoid structures. However, the heterogeneity of shapes indicates substantial plasticity of telomeres.

We addressed the question of whether telomere compaction changes upon depletion of TRF1 or TRF2. We observed no major changes in telomere density. This therefore suggests that, upon shelterin removal, telomeric DNA remains compacted. TRF2 is able to package telomeric DNA in vitro (Benarroch-Popivker et al. 2016), but the lack of this activity upon TRF2 depletion can obviously be compensated for by other factors such as nucleosomes or other proteins that remain unidentified. We also specifically analyzed the sizes of TRF2-depleted telomeres that elicited a DDR. This was possible by costaining of telomeres with the DDR markers 53BP1 and γH2AX. This analysis revealed that the majority of telomeres eliciting a DDR did not differ in size in comparison with their DDR-negative counterparts. This therefore strongly indicates that telomere decompaction is not linked to the DDR. In addition, the data suggest that t-loop unfolding does not lead to massive telomere expansions and shape changes. A small subset of DDR-positive telomeres showed a higher *R*_g_. However, these telomeres had proportionally higher numbers of localizations. Consistent with this, telomeres stained with TRF1 antibody gave similar size distributions, and, again, larger telomere foci had a higher number of localizations. As TRF2 depletion does not lead to rapid telomere elongation, the most straightforward interpretation of these results is that the large telomeres with a high number of localizations correspond to telomere clusters. However, we cannot fully exclude that the small subset of DDR-positive telomeres with a large *R*_g_ corresponds to decompacted telomeres that at the same time became more susceptible to labeling with the FISH probe and the TRF1 antibodies.

Our data indicate that decompaction of telomeres is not required for the telomeric DDR. On the other hand, we observed a high plasticity of telomere shapes. This suggests a dynamic nature of telomeres, which may facilitate protein composition changes at telomeres in response to cell cycle, cell differentiation, or stress. We therefore favor the idea that telomeres are constantly accessible to proteins, including the checkpoint machinery, that monitor their intactness. Consistent with this model are several previous observations. A rapid exchange of GFP-tagged TRF1 and TRF2 in the second to minute scale at chromosome ends was demonstrated, supporting a dynamic model for telomeres with a constant flux of its constituents (Mattern et al. 2004). Telomerase was also shown to be able to access telomeres in S phase with high frequency (Schmidt et al. 2016). Finally, Mre11, ATM, and ATR were detected at telomeres in chromatin immunoprecipitation experiments from late S phase to the G2/M transition (Verdun and Karlseder 2006), where they may promote telomerase recruitment (Lee et al. 2015; Tong et al. 2015). Thus, our study and others support the notion that telomeres are physically accessible to non-telomere-bound proteins and that telomeres do not shield chromosome ends from protein access. In this regard, telomeres may not be substantially different from other regions of the genome that are characterized by dynamic nucleosomes, allowing the scanning of genome information (Hihara et al. 2012; Ricci et al. 2015).

Our data are consistent with the accompanying study by the Zhuang and de Lange laboratories (Timashev et al. 2017) in which the size of mouse telomeres was analyzed in the presence and absence of TRF1 and TRF2. These investigators also did not find evidence that DDR requires substantial chromatin decompaction when shelterin is compromised. However, our data and conclusions are in striking contrast to the ones put forward by the Yildiz group (Bandaria et al. 2016), who proposed that intact telomeres are excluding the checkpoint proteins because of steric hindrance from the very dense packaging. In their study, TRF1-depleted or TRF2-depleted telomeres were reported to expand up to 10-fold in volume, and, in their model, only expanded telomeres became accessible to the DDR machinery. To better facilitate comparison with other works, we reanalyzed our data using the convex hull as a readout for size (Supplemental Fig. S6). We estimated the convex hull volume by computing the convex hull areas for all of our two-dimensional (2D) clusters and raised these values to the power 3/2. Considering that the volume increases with the third power of the radius, the convex hull comparisons inflated relative differences between HeLa S and HeLa L telomeres and DDR-negative and DDR-positive telomeres as expected (Supplemental Fig. S6A,B). However, in analogy to the *R*_g_ comparison, the difference in mean volume of DDR-negative and DDR-positive telomeres was driven by a small subset of very large telomere foci (Supplemental Fig. S6C). Thus, while we are not able to fully explain the discrepancies between the measurements, our study includes the following major advances. First, we developed an imaging platform that allowed the analysis of an unprecedentedly large number of telomeres, providing a very high confidence on our measurements. Second, we visualized telomeric DNA by FISH or endogenous TRF1 by IF without manipulating native telomere protein composition, whereas, in the previous study (Bandaria et al. 2016), key data were acquired by photoactivated localization microscopy (PALM) imaging of cells that overexpressed mEos2-tagged versions of TRF1 and TRF2. Third, we distinguished DDR-positive and DDR-negative telomeres and analyzed them separately, allowing us to identify and characterize the more heterogeneous populations of DDR-positive telomeres. Our technical developments set the stage to study telomere compaction in various cellular states and dissect the roles of telomeric chromatin components for telomere morphology.

## Materials and methods

### Cell culture

HeLa cell lines harboring 11-kb-long (HeLa S) and 33-kb-long (HeLa L) telomeres were described previously (Grolimund et al. 2013). Both cell lines were maintained at 37°C with 5% CO_2_ in Dulbecco's modified Eagle's medium supplemented with 10% fetal calf serum and penicillin/streptomycin.

### Telomere restriction fragment length analysis

Genomic DNA was isolated using the Wizard Genomic DNA purification kit (Promega). Genomic DNA (8 µg) was subjected to restriction digestion with HinfI and RsaI and separated by pulse-field gel electrophoresis on 1% agarose in 0.5× TBE at 5 V cm^−1^ for 16 h at 14°C with switch times ramped from 0.5 to 6 sec. The gel was dried for 2 h at 50°C, denatured with 0.8 M NaOH and 150 mM NaCl, neutralized with 0.5 M Tris-HCl (pH 7.0) and 1.5 M NaCl, prehybridized at 50°C in Church buffer (1% BSA, 1 mM EDTA, 0.5 M Na-phosphate buffer at pH 7.2, 7% SDS), and hybridized overnight at 50°C to a [^32^P]-labeled telomeric probe as described (Grolimund et al. 2013). After hybridization, the gel was rinsed in 4× SSC followed by successive 1-h washes at 50°C in 4× SSC, 4× SSC, 0.5% SDS, 2× SSC, and 0.5% SDS. The image was acquired using a FujiFilm Fluorescent Image Analyzer (FLA-3000).

The subtelomere sequence assemblies from the Riethman laboratory at the Wistar Institute (http://www.wistar.org/lab/harold-c-riethman-phd/page/subtelomere-assemblies) were used to calculate the average DNA length (419 bp) contributed by subtelomeric DNA to the telomere restriction fragments following HinfI and RsaI cleavage.

### Antibodies

The following antibodies were used: TRF1 (Abcam, ab371; a generous gift from Dr. Titia de Lange) for Western blots, TRF1 (affinity-purified rabbit antibody against recombinant TRF1 from serum no. 605 448) (Grolimund et al. 2013) for IF, TRF2 (Millipore, 05-521) for Western blots, γH2AX (Millipore, 05-636) for both Western blots and IF, hnRNPA1 (4B10; Santa Cruz Biotechnology, sc-32301) for Western blots, 53BP1 (Novus Biologicals, NB100-304) for IF, 53BP1 (Novus Biologicals, NB100-305) for Western blots, and phospho-ATM-Ser 1981 (Abcam, ab81292) for Western blots.

### Plasmids

Plasmids containing shRNAs used in this study were prepared by restriction cloning of annealed oligonucleotides into pSUPERpuro or pSUPERblast plasmid backbones (Oligoengine). The target sequences of the shRNAs were TRF1 (5′-GAATATTTGGTGATCCAAA-3′) cloned into pSuperPURO and TRF2 (5′--GCGCATGACAATAAGCAGA-3′) pSuperBLAST (Porro et al. 2014). The pLPC_TRF2_ΔBΔM plasmid (a generous gift from Dr. Titia de Lange; Addgene, plasmid 18008) was used for overexpression of TRF2ΔBΔM. The pLPC-N-MYC empty plasmid (Addgene, plasmid 12540) was used as a control.

### Transfection protocols

For depletion experiments, HeLa S cells were transfected in six-well plates at 60%–80% confluency using Lipofectamine 2000 according to the manufacturer's protocol (ThermoFisher, catalog no. 11668019). Puromycin (1 µg/mL; Invivogen, ant-pr-1) and 5 µg/mL blasticidin (Invivogen, ant-bl-1) were added to the medium 20–24 h after transfection, and the cells were expanded in 10-cm dishes. Selection with the two antibiotics was maintained for 4 d. Empty pSuperPURO and pSuperBLAST plasmids were used as controls in all of the experiments.

For overexpression of the TRF2ΔBΔM dominant-negative mutant, HeLa S cells were transfected in six-well plates at 60%–80% confluency using Lipofectamine 2000 according to the manufacturer's protocol (ThermoFisher, catalog no. 11668019) and harvested 48 h after transfection for Western blot and microscopy experiments.

For siRNA-mediated depletion of TRF1 (Supplemental Fig. S2), HeLa S cells were transfected using a standard Ca-phosphate protocol with 0.5 pmol of siRNA at 20%–30% confluency. TRF1-specific siRNAs corresponded to a mix of several siRNAs (Santa Cruz Biotechnology, sc-36722). As a control, a nontargeting siRNA against GFP was used (sequence: 5′-GCAGCACGACUUCUUCAAGUUdTdT-3′). Transfected HeLa S cells were harvested 48 h after transfection.

### Telomeric PNA-FISH

FISH staining of human telomeric DNA (Celli and de Lange 2005) was performed as follows. For the analyses performed in Figure 1, E and F, HeLa S and HeLa L cells were grown on coverslips (thickness 0.17 mm ± 0.005 mm; Carl Roth, YX04.1) to 80% confluency. For the shRNA-mediated depletion experiments (Figs. 2E, 3E,D), cells were grown on coverslips and harvested for Western blot and microscopy experiments after 4 d of selection. For the overexpression experiments and siRNA-mediated depletion experiments (Supplemental Fig. S2), cells were grown on coverslips and harvested after 48 h. After harvesting, the coverslips were washed in 1× PBS, fixed with 4% formaldehyde in 1× PBS at room temperature, permeabilized in 1× detergent solution (0.1% Triton X-100, 0.02% SDS in 1× PBS), and dehydrated with increasing amounts of ethanol (70%, 95%, and 100%). Dehydrated coverslips were then placed on slides containing 90 µL of hybridization mix [10 mM Tris-HCl at pH 7.4, 2% blocking reagent (Roche, reference no. 11096176001), 70% formamide, 0.1 µM A647-labeled (CCCTAA)_3_ PNA probe (PNA Bio, F1013)] and denatured for 3 min at 80°C in a hybridization oven. Subsequently, the hybridization was allowed to proceed for 3 h in a light-protected humidified chamber at 25°C. Coverslips were removed from the slide and washed twice for 15 min in buffer containing 70% formamide and 10 mM Tris-HCl (pH 7.4) and three times for 15 min with 0.1 M Tris-HCl (pH 7.2), 0.15 M NaCl, and 0.08% Tween-20. For DNA staining, DAPI was added to 1 µg/mL in the second wash. After the washes, coverslips were stored at 4°C in 1× PBS in the dark until imaging.

### Indirect IF and telomeric FISH (IF-FISH)

Indirect IF detection of human 53BP1 and γH2AX followed by telomeric FISH staining was performed as described with minor modifications (Celli and de Lange 2005). Cells were grown on coverslips (thickness 0.17 mm ± 0.005 mm [Carl Roth, YX04.1] for STORM imaging or 12 mm [Menzel-Glaser, CS12100] for confocal imaging) as described in the previous section. After harvesting, the coverslips were washed in 1× PBS, fixed with 4% formaldehyde in 1× PBS, and permeabilized in 1× detergent solution (0.1% Triton X-100, 0.02% SDS in 1× PBS). The slides were then preblocked in 2% BSA in 1× PBS, blocked for 30 min in 10% normal goat serum in 2% BSA and 1× PBS, incubated for 1 h at room temperature with either anti-53BP1 (1:2000 dilution) or anti-γH2AX (1:1000) antibody, and washed three times for 5 min in 2% BSA and 1× PBS. Alexa fluor 488-labeled goat anti-rabbit antibody (Thermo Fisher, A-11034) was used for detection of 53BP1 for STORM imaging experiments, and Alexa fluor 633-labeled goat anti-rabbit antibody (Thermo Fisher, A-21070) was used for confocal imaging experiments. Alexa fluor 488-labeled goat anti-mouse antibody (Thermo Fisher, A-11001) was used for detection of γH2AX for both STORM and confocal imaging experiments. After detection with the secondary antibody, the cells were washed three times with 1× PBS, post-fixed with 4% formaldehyde for 5 min, and dehydrated with increasing amounts of ethanol (70%, 95%, and 100%). Dehydrated coverslips were then processed in the same manner as described for the telomeric PNA-FISH procedure using a A647-(CCCTAA)_3_ PNA probe (PNA Bio, F1013) for STORM imaging and a Cy3-(CCCTAA)_3_ PNA probe (PNA Bio, F1002) for confocal imaging. For simultaneous detection of TRF1 and γH2AX, cells were stained as above except that dehydration and FISH steps were left out.

For the analysis of telomere dysfunction-induced foci after the IF-FISH procedure, the slides were mounted in VectaShield mounting medium (Vector Laboratories). Images were acquired using a Zeiss LSM 700 upright microscope equipped with an Axiocam MRm(B/W) camera and controlled by Zen2009 software. The images were analyzed using the Cell Counter plug-in for FIJI.

### Estimation of the ratio of telomere densities of HeLa L to HeLa S

A rough estimate of the ratio of volume densities of chromatin per telomere for HeLa L and HeLa S cells may be made from the data in Figure 1. The mean *R*_g_s for HeLa L and HeLa S were 0.088 µm ± 0.023 µm and 0.068 µm ± 0.021 µm (mean ± standard deviation), respectively. The average lengths for HeLa L and HeLa S were 33 kb and 11 kb, respectively. The ratio of the volume density of HeLa L telomeric chromatin, ρ_L_, to HeLa S telomeric chromatin, ρ_S_, is therefore

$$\frac{\rho_{L}}{\rho_{S}}=\left(\frac{N_{L}}{R_{g,L}^{3}}\right)\;\left(\frac{R_{g,S}^{3}}{N_{S}}\right)=\left[\frac{33\;\mathrm{kb}}{{(0.088\;\mu m)}^{3}}\right]\;\left[\frac{{\left(0.068\;\mu m\right)}^{3}}{11\;\mathrm{kb}}\right]\approx 1.4.$$

Due to the large sample sizes, the value for the standard error of the mean Rg is <1 nm. However, sampling bias in the microscopy measurements typically leads to a variation in the observed mean of approximately ±5 nm from experiment to experiment. Taking this as the value for the error in the Rg, the upper value for the range on the estimate is

$$\frac{\rho_{L}^{+}}{\rho_{S}}=\left[\frac{33\;\mathrm{kb}}{{(0.088-0.005\;\mu m)}^{3}}\right]\;\left[\frac{{\left(0.068+0.005\;\mu m\right)}^{3}}{11\;\mathrm{kb}}\right]\approx 2.0,$$

and the lower value is

$$\frac{\rho_{L}^{-}}{\rho_{S}}=\left[\frac{33\;\mathrm{kb}}{{(0.088+0.005\;\mu m)}^{3}}\right]\;\left[\frac{{\left(0.068-0.005\;\mu m\right)}^{3}}{11\;\mathrm{kb}}\right]\approx 0.93.$$

The final estimated value for the ratio of densities of telomeric chromatin is therefore ρ_*L*_/ρ_*S*_ = 1.4 + 0.6/−0.5.

### STORM image acquisition

STORM imaging was performed on a custom-built STORM microscope with a 100 × 100-µm^2^ FOV as described previously (Douglass et al. 2016). The large FOV of this microscope allowed for the simultaneous imaging of between ∼10 and 30 nuclei; a flat illumination pattern ensured uniform fluorophore photoswitching across the FOV. For each condition and replicate, three to five FOVs were acquired, depending on the density of the cells. For the present work, an additional laser (Coherent Sapphire, 488-nm peak emission wavelength, 50 mW) was introduced into the setup to image Alexa 488 IF. A dichroic filter (Chroma, Z488bcm) was used for beam combining, and fluorescence emission in the Alexa 488 channel was filtered with a GFP emission filter (Chroma, ET525/50m).

Individual coverslips containing fixed and labeled HeLa cells were placed in a custom-built sample holder containing 1000 µL of imaging buffer (see below) supplemented with an oxygen-scavenging system. Before each STORM acquisition, a wide-field image of the FOV was acquired: one for the Alexa 647 channel (50-msec exposure time at 1.4 mW in the objective back focal plane [BFP]) and one for the Alexa 488 channel (500-msec exposure at ∼0.1 mW in the objective BFP). For STORM acquisitions, 20,000 frames per FOV at 10-msec exposure time and zero interframe delay were acquired with ∼590 mW of 647-nm laser power in the objective BFP; only the Alexa 647 channel was acquired in STORM. A 405-nm laser light was applied at frame number 10,000 and steadily ramped upward between 0 and 4.0 mW in the objective BFP through the end of the acquisition. The 405-nm laser light was applied to return Alexa 647 fluorophores to the emitting state and achieve more complete spatial sampling.

The STORM imaging buffer with oxygen-scavenging system was described previously (Olivier et al. 2013) and uses millimolar concentrations of polyunsaturated hydrocarbon cyclooctatetraene to boost photon yields during STORM imaging. All reagents were purchased from Sigma-Aldrich. The images shown in Figure 1A were taken on an inverted Nikon N-STORM microscope with a 100×/1.49 N.A. apo TIRF objective (Nikon) and an EMCCD camera (Andor, iXon3 897). A 500-mW 640-nm laser (Coherent Sapphire) and a 100-mW 402-nm laser (Coherent Sapphire) were used to induce fluorophore photoswitching and control the switching rate, respectively. Molecule localization and drift correction (using cross-correlation) for data in Figure 1A only were performed in the Nikon NIS-Elements software version 4.30.01. Before the STORM acquisition, wide-field images of the DAPI and Cy5 channels were acquired. The probe used in this experiment was the Cy5-(CCCTAA)_3_ PNA probe (Eurogentec, PN-TC055-005), and the DNA was labeled with DAPI. The oxygen-scavenging system used for STORM imaging was glucose oxidase/catalase-based and prepared as described previously (Olivier et al. 2013).

### Filtering and cluster analysis of STORM data

The filtering and analysis pipeline used in this work consists of seven discrete steps that were applied to each FOV individually (Supplemental Fig. S1). Unless otherwise stated, analyses were performed in a custom-written Python analysis library (B-Store, versions 0.1.1 and 0.2.0; https://github.com/kmdouglass/bstore) for Python 3.5.

### Computing localizations from raw image stacks

Input data for the analysis pipeline originated from STORM acquisitions and consisted of stacks of images of single fluorescent molecules labeling the telomeric DNA. All STORM image stacks in this study contained 20,000 frames recorded at 10-msec exposure times with zero delay between each frame. Image stacks were saved to a disk during acquisition as multipage tagged image format (TIF) files; each frame was represented as a 2D array of pixels whose intensities (in analog to digital units) were stored as 16-bit integers. Square subregions that potentially contained single molecules were segmented from each frame using a peak-finding algorithm that incorporated a sCMOS camera-specific noise model and used a difference of smoothing filters followed by a local maximum filter (Huang et al. 2011). Localizations (i.e., estimates of single fluorophore positions in each camera frame) were determined with subpixel accuracy in the candidate regions using a previously described sCMOS camera-specific maximum likelihood estimator fitting algorithm (Huang et al. 2013). Both of these steps were implemented in MATLAB 2014a and CUDA 4.0. We used the values shown in Table 1 for the input parameters for the segmentation and fitting algorithms in all data sets.

**Table 1.**
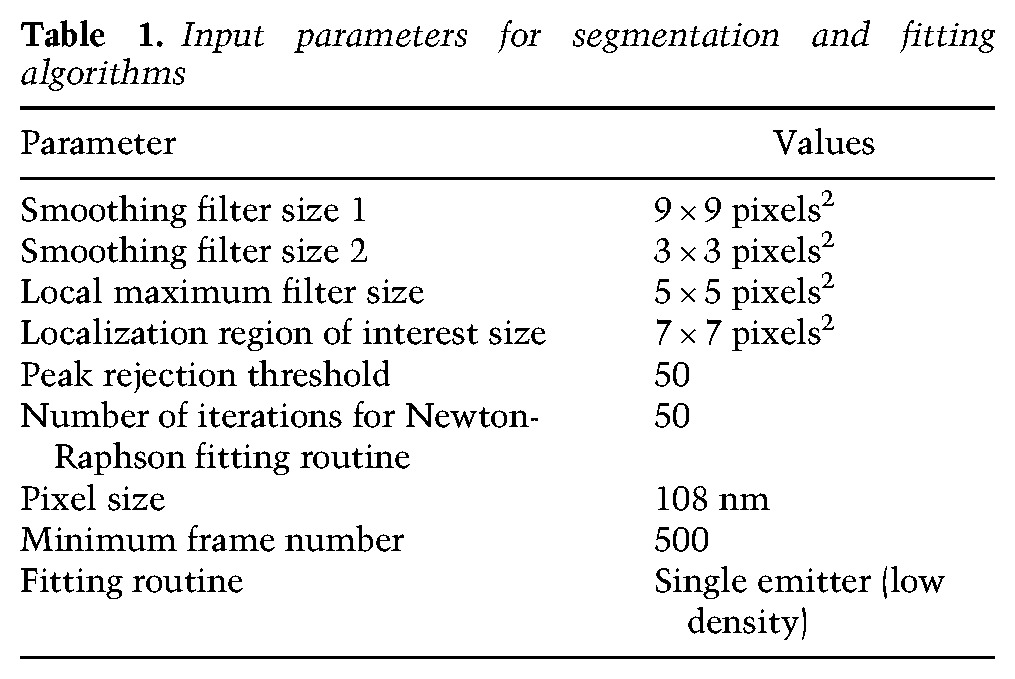
Input parameters for segmentation and fitting algorithms

Optimal values for the filter sizes and the peak threshold were determined by simultaneously varying their values and visually inspecting a small number of frames from an image stack until the majority of fluorescent spots was successfully identified. The pixel size was determined by focusing on 100-nm-diameter TetraSpeck fluorescent beads emitting light in the Alexa 647 channel (Life Technologies), depositing the beads on a coverslip, and immersing them in deionized water using the pixel size calibration routine in Micro-Manager (version 1.4.22, nightly build 2015-07-27) (Edelstein et al. 2014). Frames earlier than frame 500 were not processed because too many molecules were still emitting to allow for their accurate localization at these times. All other parameters retained their default values.

### Drift correction

Axial drift was corrected during acquisition to ∼10-nm standard deviation using a TIR laser-based active autofocus method as described previously (Douglass et al. 2016) and the pgFocus open hardware autofocus module (http://big.umassmed.edu/wiki/index.php/PgFocus). Localizations were corrected for lateral drift using 100-nm-diameter gold fiducial beads (corpuscular, 5.6 × 10^9^ particles per milliliter) that were first diluted 1:1 in 100 µg/mL poly-L-lysine (Sigma-Aldrich) to promote adhesion to the cell membrane. Prior to imaging, 200 µL of the bead suspension was pipetted onto the coverslips, allowed to sit for 5 min, and then gently washed once with phosphate-buffered saline before coverslips were immersed in STORM imaging buffer.

Localizations originating from fiducial beads were identified in the localization data sets by rendering 2D histograms with 1 × 1 µm^2^ bin sizes and manually selecting bins containing a number of localizations that was approximately equivalent to the number of frames in the image stacks. For each region, tracks of the *x* and *y* positions of the localizations versus camera frames were fit with a weighted cubic smoothing spline using a Gaussian smoothing filter for weighting whose standard deviation was typically equivalent to 200 frames and whose window size was 800 frames. These numbers were varied slightly on a case-by-case basis if spline fits were poor. The resulting splines for each fiducial track were averaged together to form a final drift trajectory and correct the localizations in that particular FOV. FOVs that contained no good fiducials were discarded from the analysis.

### Filtering and merging localizations

Localizations with precision estimated by the fitting algorithm to be >30 nm and log likelihood ratios >250 were discarded from the analysis. (The log likelihood ratio is a measure of how closely a single-molecule image resembles a 2D Gaussian point spread function [PSF] model.) The value for the localization precision filter was chosen to be approximately three times the measured localization precision (Supplemental Fig. S1); with this value, ∼99% of all localizations corresponding to a single fluorescent molecule should be retained when their emissions are well separated in time. The maximum log likelihood was selected by varying its value and observing scatter plots of localizations overlaid on the wide-field images. An optimal value struck a balance between rejecting localizations that did not overlap with any features in the wide-field images and accepting all localizations originating from the telomeres.

Because we performed 2D STORM imaging to obtain as high a localization precision as possible, we removed localizations whose fitted PSF images had widths >175 nm (standard deviation of the 2D Gaussian). This ensured that only localizations coming from a focal volume of small axial extent were retained for analysis. The average width of all localizations prior to filtering was typically ∼150 nm. After filtering, localizations were merged along the time dimension to reduce stochasticity in the spatial sampling of the telomeres due to rapid blinking of the Alexa 647 molecules, whose off-time distribution displayed two behaviors: a short pronounced peak at the origin and a long tail comprised of relatively few events (Supplemental Fig. S1). The merge radius was set to 30 nm (or three times the measured mean localization precision), and the gap time was set to one frame to balance the rapid blinking behavior against the chance to erroneously merge localizations from distinct molecules. This meant that a track of localizations could disappear and reappear for at most one frame and still be merged into a single localization. Merging was performed using a Python implementation of the Crocker-Grier tracking algorithm (Trackpy version 0.3.0; http://doi.org/10.5281/zenodo.34028).

### Spatial clustering

Localizations were spatially clustered using the DBSCAN clustering algorithm (Martin et al. 1996) from scikit-learn (version 0.17.1). The minimum number of localizations per cluster was set to eight, and the neighborhood radius was set to 90 nm. These values were determined by varying them and simultaneously observing the results of the clustering of localizations overlaid on a corresponding wide-field image. Ideal values did not erroneously group distinct clusters of localizations that originated from separate telomere signals into single clusters; ideal values also did not break up distinct clusters into multiple subclusters.

After clustering, we removed clusters with <50 localizations because these clusters very often did not overlap a feature in the wide-field images as described below.

### Alignment to wide-field images

Each set of clustered localizations was binned into a separate 2D histogram with bin side lengths of ∼22 nm. The corresponding wide-field fluorescence images in the Alexa 647 channel were upsampled five times to the same pixel size (22 nm) and cross-correlated with the localization histograms using a fast Fourier transform-based implementation (fftconvolve method from Scipy version 0.17.1) to determine and correct any offset between the localizations and the wide-field images. We typically observed an offset that was between 40 and 60 nm in each direction that was attributed to both the stage drift and the drift correction procedure described above. The calculated offsets were applied to the localizations to ensure that they were correctly overlaid on top of their corresponding features in the wide-field images in the next steps of the analysis pipeline.

Wide-field images in the Alexa 488 and Alexa 647 channels were acquired in quick succession so that stage drift between the two acquisitions was effectively zero. We therefore used the same offsets as determined above to overlay the localizations on the wide-field images from the Alexa 488 channel. A small axial displacement of the piezo stage of 0.6 µm was made between channels to correct axial chromatic aberration. Due to the large sizes of the 53BP1 and γH2AX loci, we did not observe the need to correct transverse chromatic aberrations to determine the overlap of a telomeric STORM signal with a DDR locus (Fig. 3B).

### Manual cluster rejection

To ensure that each cluster of localizations was telomeric in origin, we performed a semiautomated filtering step for every FOV. Clusters of localizations were overlaid on top of the wide-field images (after applying the offsets described above) and presented one-by-one to the analyst. The analyst chose to keep or reject each cluster based on the following criteria: (1) Clusters were located on top of a fluorescent locus. (2) Clusters were located inside a cell nucleus. (3) The shape of the cluster roughly matched the shape of the corresponding wide-field locus. After each decision, the analysis software recorded the results and automatically progressed to the next cluster. This step was performed with the custom-written Python analysis library described above.

### Manual cluster classification

For experiments in which we determined whether a 53BP1 or γH2AX signal was present at any given telomere, the manually filtered clusters of localizations were overlaid on top of the wide-field image from the Alexa 488 channel (applying the lateral offset as described above). Each telomere was then manually classified into one of three groups: (1) no overlap of the cluster with an Alexa 488 locus, (2) partial spatial overlap of the cluster with an Alexa 488 locus, and (3) complete spatial overlap of the cluster with an Alexa 488 locus. Once again, the custom software for this semiautomated analysis is at the URL above.

### Data availability

All original data are available from the Dryad Digital Repository (http://dx.doi.org/10.5061/dryad.h1157).

## Supplementary Material

Supplemental Material

## References

[VANCEVSKAGAD294082C1] Bandaria JN, Qin P, Berk V, Chu S, Yildiz A. 2016 Shelterin protects chromosome ends by compacting telomeric chromatin. Cell 164: 735–746. 26871633 10.1016/j.cell.2016.01.036 PMC4762449

[VANCEVSKAGAD294082C2] Bartocci C, Diedrich JK, Ouzounov I, Li J, Piunti A, Pasini D, Yates JR III, Lazzerini Denchi E. 2014 Isolation of chromatin from dysfunctional telomeres reveals an important role for Ring1b in NHEJ-mediated chromosome fusions. Cell Rep 7: 1320–1332. 24813883 10.1016/j.celrep.2014.04.002 PMC4054697

[VANCEVSKAGAD294082C3] Baumann P, Cech T. 2001 Pot1, the putative telomere end-binding protein in fission yeast and humans. Science 292: 1171–1175. 11349150 10.1126/science.1060036

[VANCEVSKAGAD294082C4] Benarroch-Popivker D, Pisano S, Mendez-Bermudez A, Lototska L, Kaur P, Bauwens S, Djerbi N, Latrick CM, Fraisier V, Pei B, 2016 TRF2-mediated control of telomere DNA topology as a mechanism for chromosome-end protection. Mol Cell 61: 274–286. 26774283 10.1016/j.molcel.2015.12.009 PMC5001171

[VANCEVSKAGAD294082C6] Celli GB, de Lange T. 2005 DNA processing is not required for ATM-mediated telomere damage response after TRF2 deletion. Nat Cell Biol 7: 712–718. 15968270 10.1038/ncb1275

[VANCEVSKAGAD294082C7] Cristofari G, Lingner J. 2006 Telomere length homeostasis requires that telomerase levels are limiting. EMBO J 25: 565–574. 16424902 10.1038/sj.emboj.7600952 PMC1383536

[VANCEVSKAGAD294082C8] Dejardin J, Kingston RE. 2009 Purification of proteins associated with specific genomic Loci. Cell 136: 175–186. 19135898 10.1016/j.cell.2008.11.045 PMC3395431

[VANCEVSKAGAD294082C9] de Lange T. 2005 Shelterin: the protein complex that shapes and safeguards human telomeres. Genes Dev 19: 2100–2110. 16166375 10.1101/gad.1346005

[VANCEVSKAGAD294082C10] de Lange T. 2009 How telomeres solve the end-protection problem. Science 326: 948–952. 19965504 10.1126/science.1170633 PMC2819049

[VANCEVSKAGAD294082C11] Denchi EL, de Lange T. 2007 Protection of telomeres through independent control of ATM and ATR by TRF2 and POT1. Nature 448: 1068–1071. 17687332 10.1038/nature06065

[VANCEVSKAGAD294082C12] Denchi EL, Sfeir A. 2016 Stop pulling my strings-what telomeres taught us about the DNA damage response. Nat Rev Mol Cell Biol 17: 364–378. 27165790 10.1038/nrm.2016.43 PMC5385261

[VANCEVSKAGAD294082C14] Doksani Y, Wu JY, de Lange T, Zhuang X. 2013 Super-resolution fluorescence imaging of telomeres reveals TRF2-dependent T-loop formation. Cell 155: 345–356. 24120135 10.1016/j.cell.2013.09.048 PMC4062873

[VANCEVSKAGAD294082C15] Douglass KM, Sieben C, Archetti A, Lambert A, Manley S. 2016 Super-resolution imaging of multiple cells by optimised flat-field epi-illumination. Nat Photonics 10: 705–708. 27818707 10.1038/nphoton.2016.200 PMC5089541

[VANCEVSKAGAD294082C16] Edelstein AD, Tsuchida MA, Amodaj N, Pinkard H, Vale RD, Stuurman N. 2014 Advanced methods of microscope control using muManager software. J Biol Methods 1: e10. 25606571 10.14440/jbm.2014.36 PMC4297649

[VANCEVSKAGAD294082C17] Griffith JD, Comeau L, Rosenfield S, Stansel RM, Bianchi A, Moss H, de Lange T. 1999 Mammalian telomeres end in a large duplex loop. Cell 97: 503–514. 10338214 10.1016/s0092-8674(00)80760-6

[VANCEVSKAGAD294082C18] Grolimund L, Aeby E, Hamelin R, Armand F, Chiappe D, Moniatte M, Lingner J. 2013 A quantitative telomeric chromatin isolation protocol identifies different telomeric states. Nat Commun 4: 2848. 24270157 10.1038/ncomms3848

[VANCEVSKAGAD294082C19] Hihara S, Pack CG, Kaizu K, Tani T, Hanafusa T, Nozaki T, Takemoto S, Yoshimi T, Yokota H, Imamoto N, 2012 Local nucleosome dynamics facilitate chromatin accessibility in living mammalian cells. Cell Rep 2: 1645–1656. 23246002 10.1016/j.celrep.2012.11.008

[VANCEVSKAGAD294082C20] Huang F, Schwartz SL, Byars JM, Lidke KA. 2011 Simultaneous multiple-emitter fitting for single molecule super-resolution imaging. Biomed Opt Expr 2: 1377–1393. 10.1364/BOE.2.001377 PMC3087594 21559149

[VANCEVSKAGAD294082C21] Huang F, Hartwich TM, Rivera-Molina FE, Lin Y, Duim WC, Long JJ, Uchil PD, Myers JR, Baird MA, Mothes W, 2013 Video-rate nanoscopy using sCMOS camera-specific single-molecule localization algorithms. Nat Methods 10: 653–658. 23708387 10.1038/nmeth.2488 PMC3696415

[VANCEVSKAGAD294082C22] Karlseder J, Hoke K, Mirzoeva OK, Bakkenist C, Kastan MB, Petrini JH, de Lange T. 2004 The telomeric protein TRF2 binds the ATM kinase and can inhibit the ATM-dependent DNA damage response. PLoS Biol 2: E240. 15314656 10.1371/journal.pbio.0020240 PMC509302

[VANCEVSKAGAD294082C23] Lambert TJ, Waters JC. 2017 Navigating challenges in the application of superresolution microscopy. J Cell Biol 216: 53–63. 27920217 10.1083/jcb.201610011 PMC5223610

[VANCEVSKAGAD294082C24] Lee JH, Paull TT. 2005 ATM activation by DNA double-strand breaks through the Mre11–Rad50–Nbs1 complex. Science 308: 551–554. 15790808 10.1126/science.1108297

[VANCEVSKAGAD294082C25] Lee SS, Bohrson C, Pike AM, Wheelan SJ, Greider CW. 2015 ATM kinase is required for telomere elongation in mouse and human cells. Cell Rep 13: 1623–1632. 26586427 10.1016/j.celrep.2015.10.035 PMC4663052

[VANCEVSKAGAD294082C26] Martin ES, Kriegel H-P, Sander J, Xu X. 1996 A density-based algorithm for discovering clusters in large spatial databases with noise. Kdd 96: 226–231.

[VANCEVSKAGAD294082C27] Mattern KA, Swiggers SJ, Nigg AL, Lowenberg B, Houtsmuller AB, Zijlmans JM. 2004 Dynamics of protein binding to telomeres in living cells: implications for telomere structure and function. Mol Cell Biol 24: 5587–5594. 15169917 10.1128/MCB.24.12.5587-5594.2004 PMC419875

[VANCEVSKAGAD294082C28] Okamoto K, Bartocci C, Ouzounov I, Diedrich JK, Yates JR III, Denchi EL. 2013 A two-step mechanism for TRF2-mediated chromosome-end protection. Nature 494: 502–505. 23389450 10.1038/nature11873 PMC3733551

[VANCEVSKAGAD294082C29] Olivier N, Keller D, Gonczy P, Manley S. 2013 Resolution doubling in 3D-STORM imaging through improved buffers. PLoS One 8: e69004. 23874848 10.1371/journal.pone.0069004 PMC3714239

[VANCEVSKAGAD294082C30] Poon SS, Lansdorp PM. 2001 Quantitative fluorescence in situ hybridization (Q-FISH). Curr Protoc Cell Biol 12: 18.4.1–18.4.21. 10.1002/0471143030.cb1804s12 18228343

[VANCEVSKAGAD294082C31] Porro A, Feuerhahn S, Delafontaine J, Riethman H, Rougemont J, Lingner J. 2014 Functional characterization of the TERRA transcriptome at damaged telomeres. Nat Commun 5: 5379. 25359189 10.1038/ncomms6379 PMC4264578

[VANCEVSKAGAD294082C32] Ricci MA, Manzo C, Garcia-Parajo MF, Lakadamyali M, Cosma MP. 2015 Chromatin fibers are formed by heterogeneous groups of nucleosomes in vivo. Cell 160: 1145–1158. 25768910 10.1016/j.cell.2015.01.054

[VANCEVSKAGAD294082C33] Rust MJ, Bates M, Zhuang X. 2006 Sub-diffraction-limit imaging by stochastic optical reconstruction microscopy (STORM). Nat Methods 3: 793–795. 16896339 10.1038/nmeth929 PMC2700296

[VANCEVSKAGAD294082C34] Schmidt JC, Zaug AJ, Cech TR. 2016 Live cell imaging reveals the dynamics of telomerase recruitment to telomeres. Cell 166: 1188–1197 e1189. 27523609 10.1016/j.cell.2016.07.033 PMC5743434

[VANCEVSKAGAD294082C35] Sfeir A, Kosiyatrakul ST, Hockemeyer D, MacRae SL, Karlseder J, Schildkraut CL, de Lange T. 2009 Mammalian telomeres resemble fragile sites and require TRF1 for efficient replication. Cell 138: 90–103. 19596237 10.1016/j.cell.2009.06.021 PMC2723738

[VANCEVSKAGAD294082C36] Timashev LA, Babcock H, Zhuang X, de Lange T. 2017 The DDR at telomeres lacking intact shelterin does not require substantial chromatin decompaction. Genes Dev (this issue) 10.1101/gad.294108.116. PMC5393053 28381412

[VANCEVSKAGAD294082C37] Tong AS, Stern JL, Sfeir A, Kartawinata M, de Lange T, Zhu XD, Bryan TM. 2015 ATM and ATR signaling regulate the recruitment of human telomerase to telomeres. Cell Rep 13: 1633–1646. 26586433 10.1016/j.celrep.2015.10.041 PMC4662887

[VANCEVSKAGAD294082C38] Uziel T, Lerenthal Y, Moyal L, Andegeko Y, Mittelman L, Shiloh Y. 2003 Requirement of the MRN complex for ATM activation by DNA damage. EMBO J 22: 5612–5621. 14532133 10.1093/emboj/cdg541 PMC213795

[VANCEVSKAGAD294082C39] van Steensel B, Smogorzewska A, de Lange T. 1998 TRF2 protects human telomeres from end-to-end fusions. Cell 92: 401–413. 9476899 10.1016/s0092-8674(00)80932-0

[VANCEVSKAGAD294082C40] Verdun RE, Karlseder J. 2006 The DNA damage machinery and homologous recombination pathway act consecutively to protect human telomeres. Cell 127: 709–720. 17110331 10.1016/j.cell.2006.09.034

[VANCEVSKAGAD294082C41] Zimmermann M, Kibe T, Kabir S, de Lange T. 2014 TRF1 negotiates TTAGGG repeat-associated replication problems by recruiting the BLM helicase and the TPP1/POT1 repressor of ATR signaling. Genes Dev 28: 2477–2491. 25344324 10.1101/gad.251611.114 PMC4233241

[VANCEVSKAGAD294082C42] Zou L, Elledge SJ. 2003 Sensing DNA damage through ATRIP recognition of RPA–ssDNA complexes. Science 300: 1542–1548. 12791985 10.1126/science.1083430

