# Supplementary material for: The telomeric DNA damage response occurs in the absence of chromatin decompaction
Source: Genes Dev. 2017 Mar 15;31(6):567–77. doi: 10.1101/gad.294082.116 (PMC5393052; doi:10.1101/gad.294082.116)
Supplement: Supplemental Material [file supp_31_6_567__index.html]

The telomeric DNA damage response occurs in the absence of chromatin decompaction — Supplemental Material 

# The telomeric DNA damage response occurs in the absence of chromatin decompaction

## Supplemental Material

- Supplemental\_Fig\_S1.pdf
- Supplemental\_Fig\_S3.pdf
- Supplemental\_Fig\_S6.pdf
- Supplemental\_Fig\_S2.pdf
- Supplemental\_Fig\_S4.pdf
- Supplemental\_Fig\_S5.pdf
