# Supplementary material for: The telomeric DNA damage response occurs in the absence of chromatin decompaction
Source: Genes Dev. 2017 Mar 15;31(6):567–77. doi: 10.1101/gad.294082.116 (PMC5393052; doi:10.1101/gad.294082.116)
Supplement: Supplemental Material [file supp_gad.294082.116_Supplemental_Fig_S1.pdf]

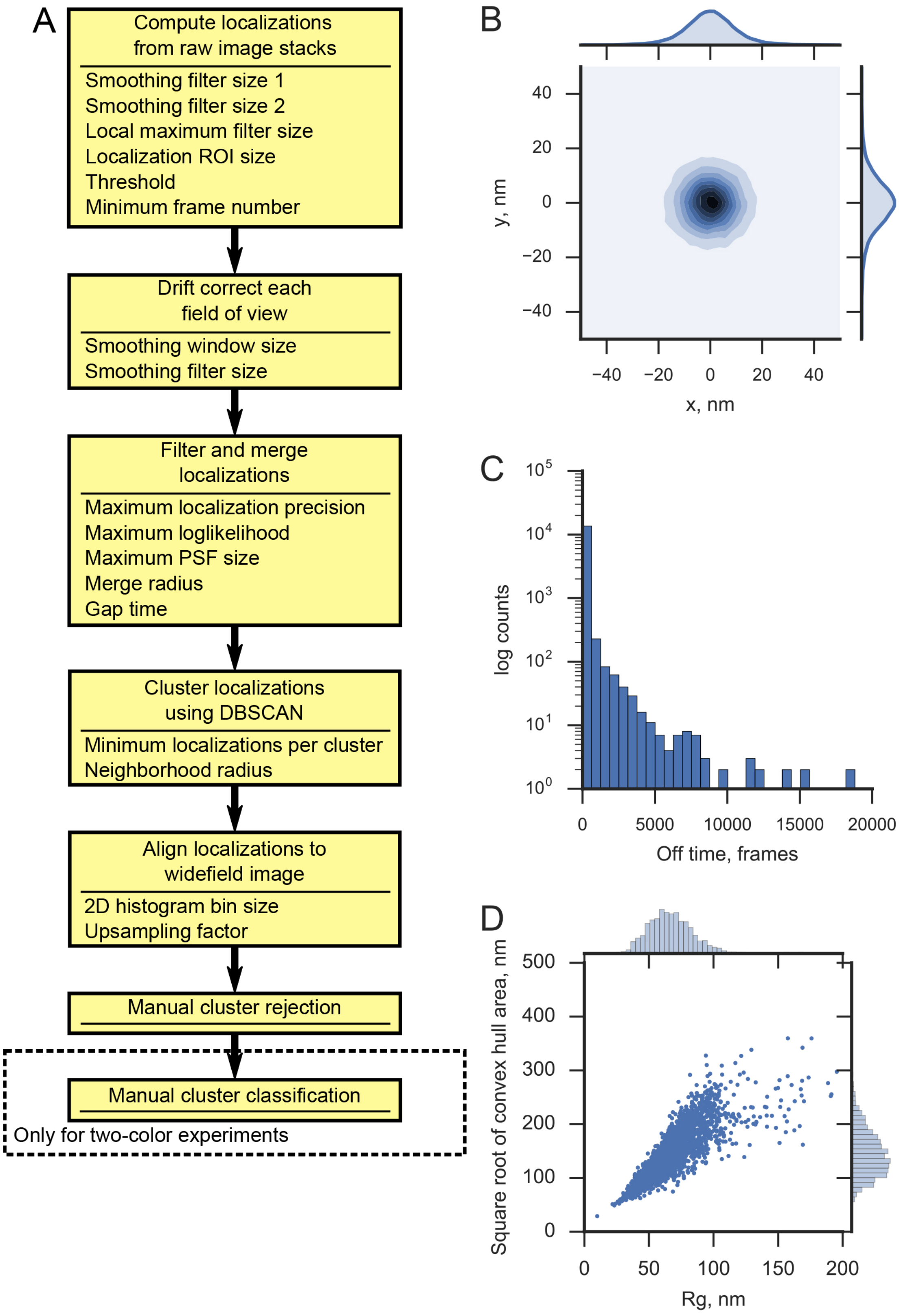

Supplemental Figure S1: Processing pipeline and supplemental data for STORM cluster analyses.

(A) The processing pipeline consists of six (possibly seven) steps that require setting values for the parameters described in the text. (B) 2D kernel density estimate of overlapped clusters of localizations from individual AlexaFluor 647 dye molecules conjugated to PNA oligonucleotides (5'-CCCTAA-3')$_3$, sparsely distributed on a coverslip in imaging buffer, and imaged using the STORM acquisition parameters described in the text. The 10 nm localization precision is the standard deviation of the distribution in each direction. (C) Off-time distribution of the AlexaFluor 647 molecules shows a strong propensity of the dye to blink rapidly. (D) The convex hull area and the radius of gyration (Rg) both measure the size of a cluster of localizations. Data are taken from HeLa S telomeres (Figure 1).
