# Supplementary material for: The telomeric DNA damage response occurs in the absence of chromatin decompaction
Source: Genes Dev. 2017 Mar 15;31(6):567–77. doi: 10.1101/gad.294082.116 (PMC5393052; doi:10.1101/gad.294082.116)
Supplement: Supplemental Material [file supp_gad.294082.116_Supplemental_Fig_S3.pdf]

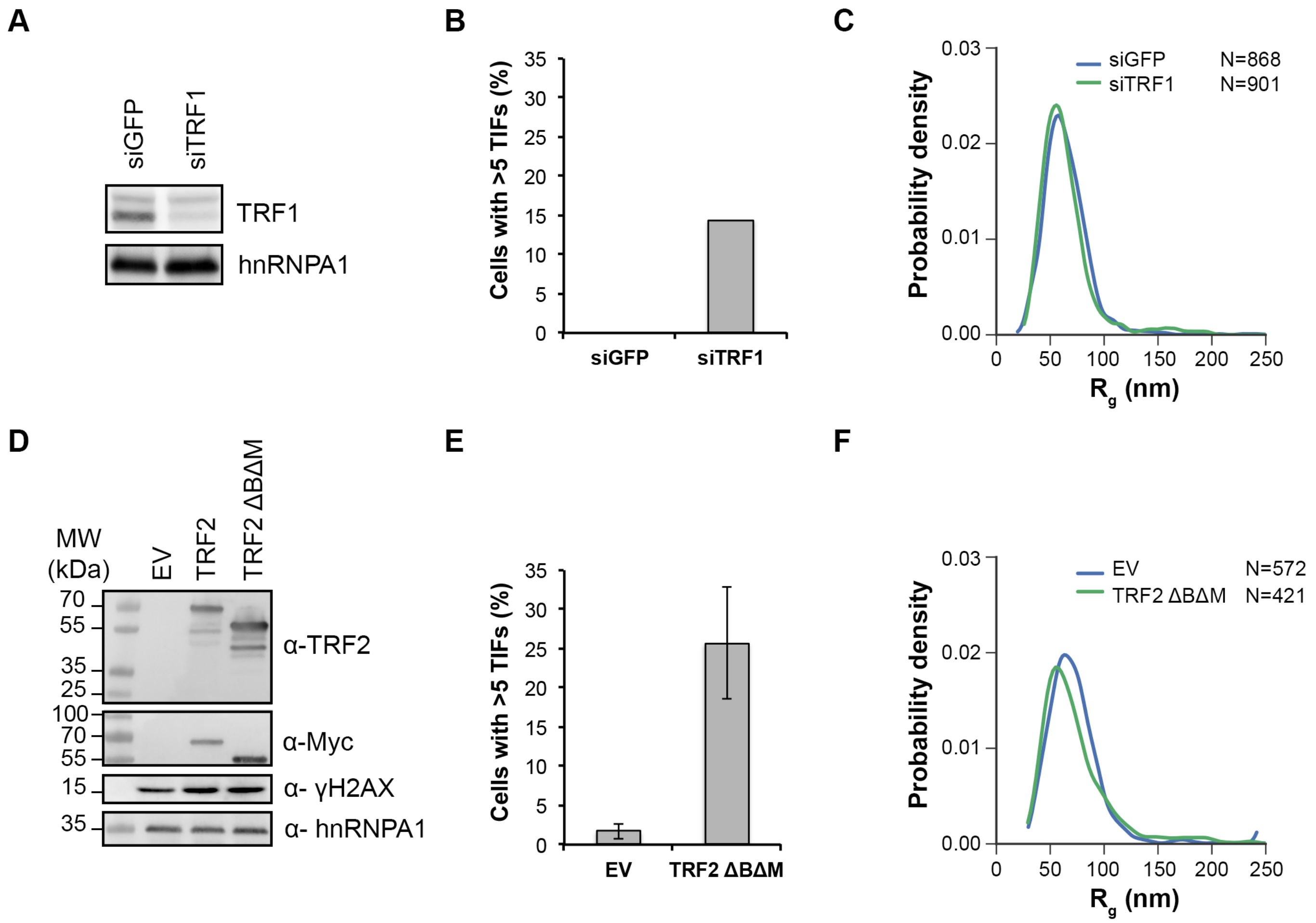

Supplemental Figure S3: Depletion of shelterin proteins TRF1 and TRF2 does not affect telomere size in HeLaS cells.

(A) Western Blot analysis of TRF1 and hnRNPA1 in HeLaS cells transfected with the indicated siRNAs (siGFP and siTRF1). (B) Quantification of the number of cells containing >5 telomere dysfunction induced foci (TIFs) detected as in Figure2B. Data represent mean of 1 independent experiment (>200 cells/condition/experiment). (C) Distributions of Rg of telomeric $(CCCTAA)_3$-FISH labeled samples obtained by analysis of STORM data. (D) Western Blot analysis of TRF2, Myc, γH2AX and hnRNPA1 in HeLaS cells transfected with TRF2 ΔBΔM and empty vector (EV) control. (E) Quantification of the number of cells containing >5 telomere dysfunction induced foci (TIFs) detected as in Figure2B. Data represent mean of 2 independent experiments (>190 cells/condition/experiment). (F) The same as in (C) but for TRF2 ΔBΔM transfected cells.
