# Supplementary material for: The telomeric DNA damage response occurs in the absence of chromatin decompaction
Source: Genes Dev. 2017 Mar 15;31(6):567–77. doi: 10.1101/gad.294082.116 (PMC5393052; doi:10.1101/gad.294082.116)
Supplement: Supplemental Material [file supp_gad.294082.116_Supplemental_Fig_S6.pdf]

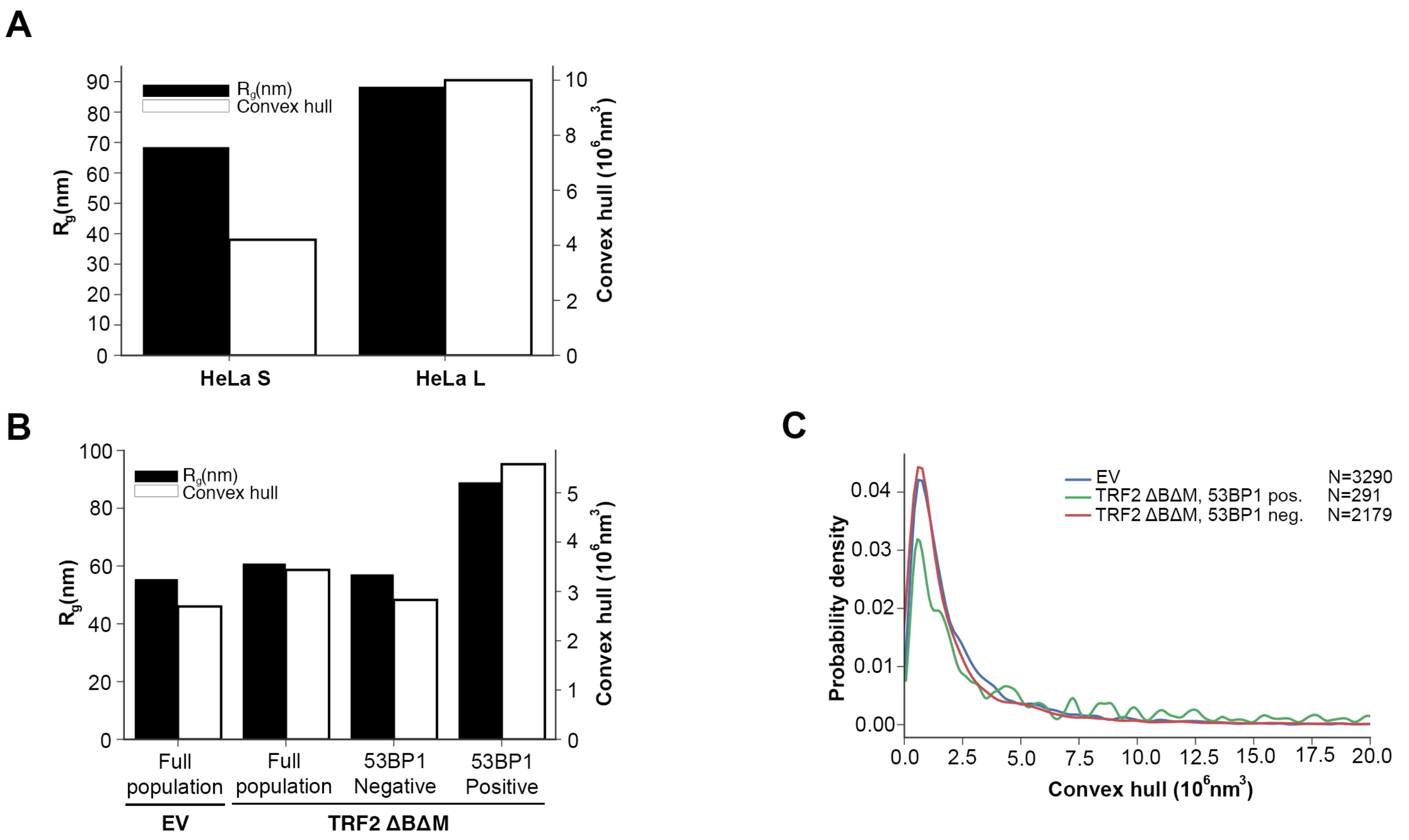

Supplemental Figure S6: Comparison of radius of gyration and convex hull measurements of telomere size.

(A) Average radius of gyration (R_g) and average convex hull volume of telomeric $(CCCTAA)_3$-FISH labeled HeLaS and HeLaL cell lines obtained by analysis of STORM data shown in Figure 1. (B) Average radius of gyration (R_g) and average convex hull volume of telomeric $(CCCTAA)_3$-FISH and 53BP1 labeled samples obtained by analysis of STORM data in HeLaS transfected with TRF2 ΔBΔM and empty vector control (EV). Data shown is from experiment in Supplementary Figure S4. (C) Representative convex hull distributions of telomeric $(CCCTAA)_3$-FISH and 53BP1 labeled samples obtained by analysis of STORM data in HeLaS cells transfected with TRF2 ΔBΔM and empty vector control (EV).
