# Supplementary material for: The telomeric DNA damage response occurs in the absence of chromatin decompaction
Source: Genes Dev. 2017 Mar 15;31(6):567–77. doi: 10.1101/gad.294082.116 (PMC5393052; doi:10.1101/gad.294082.116)
Supplement: Supplemental Material [file supp_gad.294082.116_Supplemental_Fig_S2.pdf]

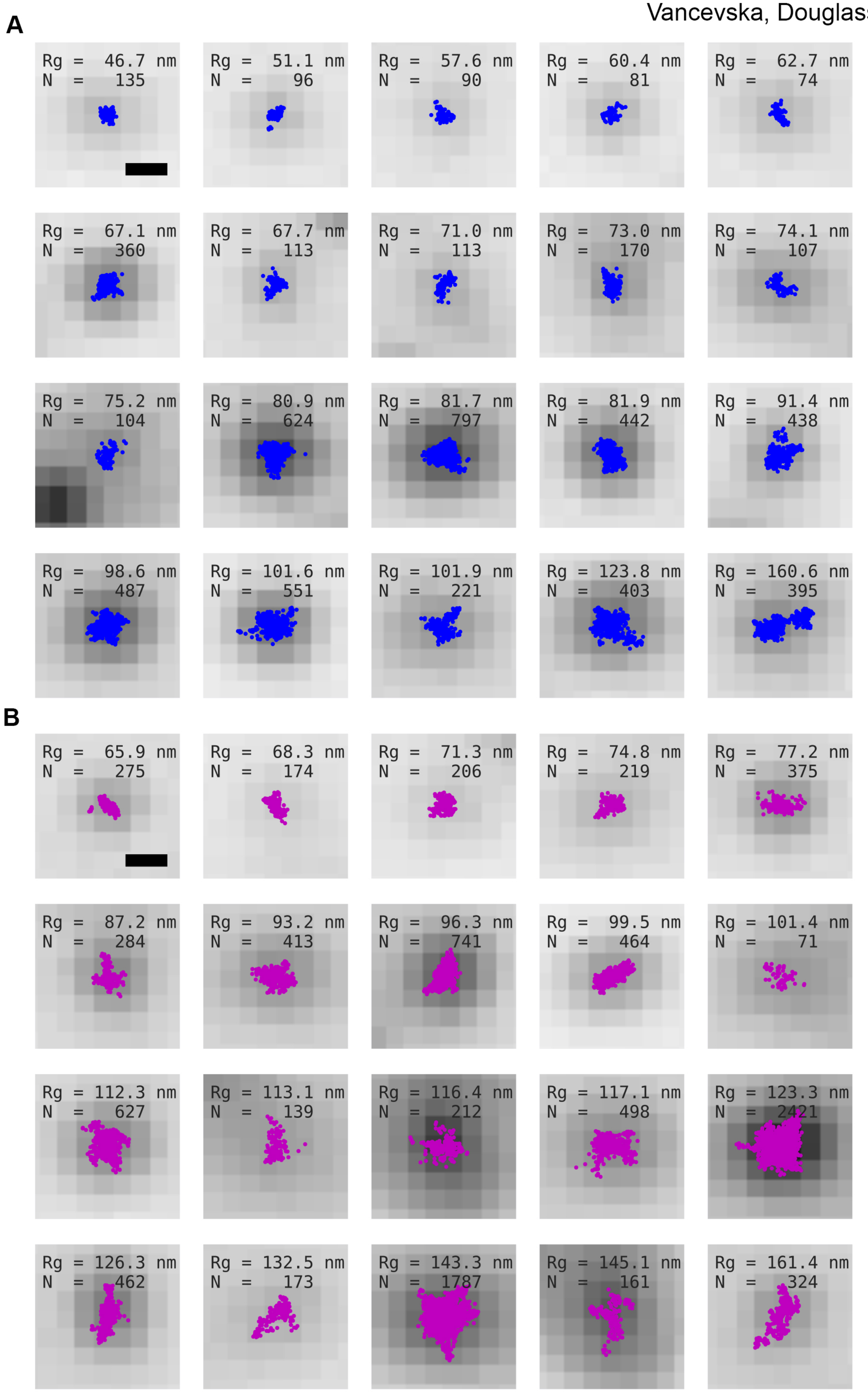

Supplemental Figure S2: Visual comparison of telomeres from populations with differeent mean lengths.

(A) Scatter plots of localizations overlaid on the corresponding widefield images of twenty telomeres drawn randomly from the HeLa S population of Figure1. (B) Twenty clusters randomly drawn from the HeLa L population of Figure 1. Rg: radius of gyration. N: number of localizations. Scale bars: 250 nm.
