# Supplementary material for: The telomeric DNA damage response occurs in the absence of chromatin decompaction
Source: Genes Dev. 2017 Mar 15;31(6):567–77. doi: 10.1101/gad.294082.116 (PMC5393052; doi:10.1101/gad.294082.116)
Supplement: Supplemental Material [file supp_gad.294082.116_Supplemental_Fig_S4.pdf]

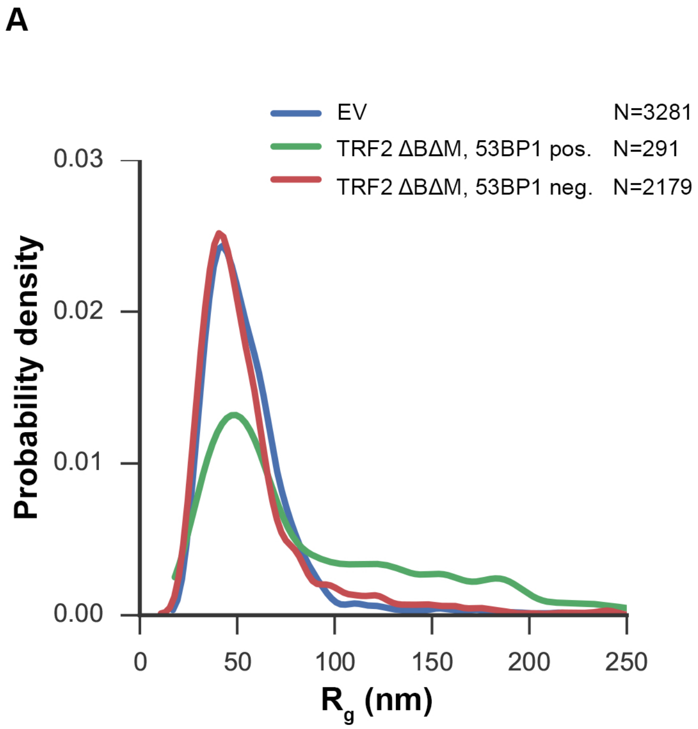

Supplemental Figure S4: Selection of DDR positive telomeres of TRF2ΔBΔM transfected HeLa S cells with two markers (53BP1 and γ-H2AX) reveals increase in telomeres size in only a small subset of telomeres.

(A) Representative Rg distributions of telomeric $(CCCTAA)_3$-FISH and 53BP1 labeled samples obtained by analysis of STORM data in HeLaS cells transfected with TRF2 ΔBΔM and empty vector control (EV). (B) Representative Rg distributions of telomeric $(CCCTAA)_3$-FISH and γH2AX labeled samples obtained by analysis of STORM data in HeLaS transfected with TRF2 ΔBΔM and empty vector control (EV).
