# Supplementary material for: The telomeric DNA damage response occurs in the absence of chromatin decompaction
Source: Genes Dev. 2017 Mar 15;31(6):567–77. doi: 10.1101/gad.294082.116 (PMC5393052; doi:10.1101/gad.294082.116)
Supplement: Supplemental Material [file supp_gad.294082.116_Supplemental_Fig_S5.pdf]

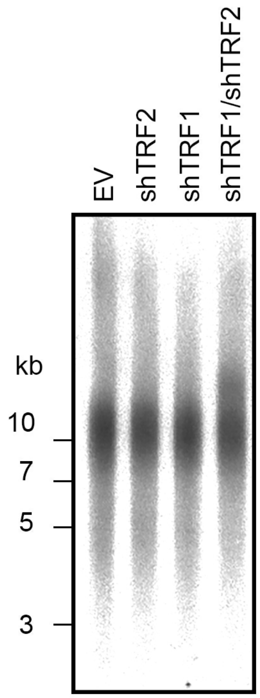

Supplemental Figure S5: Telomere restriction fragment (TRF) length analysis of HeLaS cells depleted for TRF1 and TRF2**.**
